# The Prognostic Value of Tumor-Infiltrating Neutrophils in Gastric Adenocarcinoma after Resection

**DOI:** 10.1371/journal.pone.0033655

**Published:** 2012-03-19

**Authors:** Jing-jing Zhao, Ke Pan, Wei Wang, Ju-gao Chen, Yan-heng Wu, Lin Lv, Jian-jun Li, Yi-bing Chen, Dan-dan Wang, Qiu-zhong Pan, Xiao-dong Li, Jian-chuan Xia

**Affiliations:** 1 State Key Laboratory of Oncology in Southern China and Department of Experimental Research, Sun Yat-sen University Cancer Center, Guangzhou, People's Republic of China; 2 Department of Gastric and Pancreatic Surgery, Sun Yat-sen University Cancer Center, Guangzhou, People's Republic of China; 3 Department of Thoracic Oncology, Sun Yat-sen University Cancer Center, Guangzhou, People's Republic of China; 4 First Affiliated Hospital of Jinan University, Jinan University, Guangzhou, People's Republic of China; Tufts University, United States of America

## Abstract

**Background:**

Several pieces of evidence indicate that tumor-infiltrating neutrophils (TINs) are correlated to tumor progression. In the current study, we explore the relationship between TINs and clinicopathological features of gastric adenocarcinoma patients. Furthermore, we investigated the prognostic value of TINs.

**Patients and Methods:**

The study was comprised of two groups, training group (115 patients) and test group (97 patients). Biomarkers (intratumoral CD15+ neutrophils) were assessed by immunohistochemistry. The relationship between clinicopathological features and patient outcome were evaluated using Cox regression and Kaplan-Meier analysis.

**Results:**

Immunohistochemical detection showed that the tumor-infiltrating neutrophils (TINs) in the training group ranged from 0.00–115.70 cells/high-power microscopic field (HPF) and the median number was 21.60 cells/HPF. Based on the median number, the patients were divided into high and low TINs groups. Chi-square test analysis revealed that the density of CD15+ TINs was positively associated with lymph node metastasis (p = 0.024), distance metastasis (p = 0.004) and UICC (International Union Against Cancer) staging (p = 0.028). Kaplan-Meier analysis showed that patients with a lower density of TINs had a better prognosis than patients with a higher density of TINs (p = 0.002). Multivariate Cox's analysis showed that the density of CD15+ TINs was an independent prognostic factor for overall survival of gastric adenocarcinoma patients. Using another 97 patients as a test group and basing on the median number of TINs (21.60 cells/HPF) coming from the training group, Kaplan-Meier analysis also showed that patients with a lower density of TINs had a better prognosis than patients with a higher density of TINs (p = 0.032). The results verify that the number of CD15+ TINs can predict the survival of gastric adenocarcinoma surgical patients.

**Conclusions:**

The presence of CD15+ TINs is an independent and unfavorable factor in the prognosis of gastric adenocarcinoma patients. Targeting CD15+ TINs may be a potential intervenient therapy in the future.

## Introduction

Gastric carcinoma is one of the most common malignancies, which account for a significant number of cancer-related deaths in the world [1]–[2]. Gastric adenocarcinoma is the most common type of gastric carcinoma. Despite the great advancement in diagnosis and treatment modalities, especially surgical and targeted therapies, the survival rate remains very low at a rate of 5 years [1]. Increasing evidence suggests that inflammation is the seventh hallmark of cancer [3]. Leukocytes that infiltrated the tumor promote tumor angiogenesis, growth and invasion [4]–[7]. The type, density, and location of tumor-infiltrating immune cells in the local microenvironment have been linked to the clinical outcome of various cancer types, including gastric cancer [5], [8]–[10]. Yuan et al. showed that the elevated expression of Foxp3 in tumor-infiltrating Treg cells suppressed T-cell proliferation and contributed to gastric cancer progression in a COX-2-dependent manner [11]. A study by Zhang et al. indicated that Th17 cells may contribute to gastric cancer pathogenesis [2]. Chen et al. also demonstrated that the expression levels of IL-17 in the tumor were an independent prognostic indicator in gastric adenocarcinoma patients [12].

Neutrophils represent the 50%–70% fraction of total circulating leukocytes [13]–[14]. Several pieces of evidence showed that neutrophils promoted cancer cell migration and invasion [15] as well as tumor-induced angiogenesis [16]–[18]. The presence of intratumoral neutrophils is a poor independent prognostic factor in localized renal cell carcinoma [19]. Because the role of CD15+ TINs in gastric adenocarcinoma has not been previously elucidated, we investigated the level of CD15+ TINs by immunohistochemistry and its relationship with clinicopathological features in the current study. Furthermore, we evaluated its prognostic value to post-resection survival in gastric adenocarcinoma patients.

## Results

### Immunohistochemical characteristics and association of intratumoral CD15+ TINs with clinical and histopathologic variables in the training group

**Figure 1:**
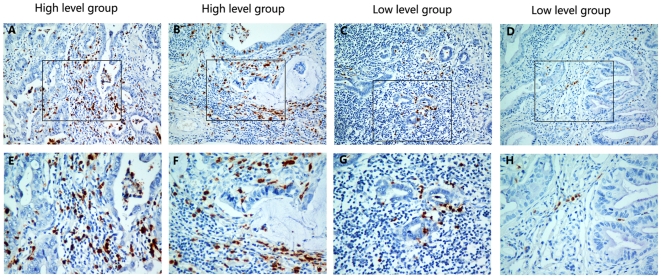
The level of CD15+ tumor-infiltrating neutrophils (TINs) in gastric adenocarcinoma surgical specimens shown by immunohistochemistry. **(A), (B), (E) and (F)**: High density of CD15+ TINs. **(C), (D), (G) and (H)**: Low density of CD15+ TINs. Original magnification: A–D×200; E–H×400.

Infiltrations of neutrophils were identified in the intratumoral stroma. Our results show variation in the level of tumor infiltrating neutrophils (TINs) (Fig. 1). In the training group which had115 cases, the number of CD15+ TINs ranged from 0.00–115.70 cells/HPF, and the median number was 21.60 cells/HPF.

To investigate the association between the density of CD15+ TINs and the clinical features in gastric adenocarcinoma patients, the median density of CD15+ TINs (21.60 cells/HPF) was used to separate the patients in the training group into high and low TINs groups (Table 1). Thus, the patients whose CD15+ TINs density above 21.60 cells/HPF were defined as high level group, and the rest were defined as low level group. The density of CD15+ TINs was positively associated with lymph node metastasis (p = 0.024), distance metastasis (p = 0.004) and UICC staging (p = 0.028). No correlations were found between the density of CD15+ TINs and the tumor size (p = 0.191), the depth of invasion (p = 0.823), the histologic grade (p = 0.322), the gender (p = 0.092) and the age (p = 0.928).

**Table 1:** Relationship between CD15+TINs and clinicopathological features of gastric adenocarcinoma patients in the training group.

Clinicopathologic variables	number of each group	CD15+TINs		*p* value
		low	high	
**All cases**	115	57	58	
**Age(years)**				0.928
<60	59	29	30	
≧60	56	28	28	
**Gender**				0.092
Male	74	41	33	
Female	41	16	25	
**Tumor size(cm)**				0.191
<4	32	19	13	
≧4	83	38	45	
**Depthof invasion**				0.823
T1–T2	17	8	9	
T3–T4	98	49	49	
**UICC staging**				0.028^a^
I–II	43	27	16	
III–IV	72	30	42	
**Lymph node metastasis**				0.024^a^
N0	37	24	13	
N1–N3	78	33	45	
**Distance metastasis**				0.004^a^
no	107	57	50	
yes	8	0	8	
**Histologic grade**				0.322
well	27	10	7	
moderate	29	16	13	
poor	59	31	28	

a *p* value<0.05.

### Correlation between CD15+ TINs and survival of patients with gastric adenocarcinoma in the training group

**Figure 2:**
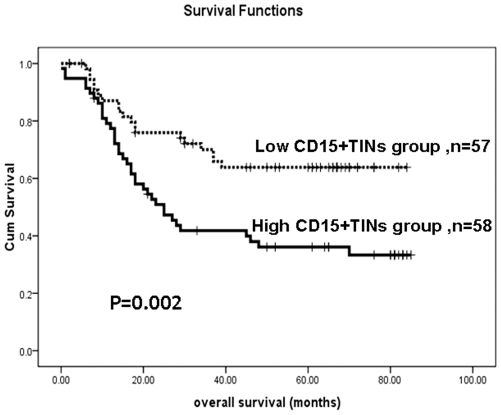
The Kaplan–Meier survival analysis of gastric adenocarcinoma patients (n = 115) in the training group with a low density of CD15+ TINs (n = 57) and a high density of CD15+ TINs (n = 58). Based on the median number of CD15+ TINs (21.60 cells/HPF) in the 115 cases, the patients were divided into two groups: high CD15+ TINs group (≧21.60 cells/HPF) and low CD15+ TINs group CD15+ TINs (<21.60 cells/HPF). The survival rate of patients with a high density of CD15+ infiltrating neutrophils was significantly lower than that of patients with a low density of CD15+ infiltrating neutrophils (Log rank test, p = 0.002).

The prognostic value of CD15+ TINs in the overall survival of gastric adenocarcinoma patients was evaluated between patients with a high or low density of infiltrating CD15+ neutrophils. The high density of CD15+TINs was significantly associated with poor prognosis of gastric adenocarcinoma patients using the Kaplan–Meier curve analysis. Gastric adenocarcinoma patients with a high density of CD15+ TINs had significantly lower overall survival rates compared to the overall survival rate of patients with a low density of CD15+ TINs (Fig. 2, p = 0.002, long rank test).

### Univariate and multivariate analyses of prognostic variables in gastric adenocarcinoma patients

To identify the variables of potential prognostic significance in the patients with gastric adenocarcinoma, univariate and multivariate analyses were further evaluated using the Cox proportional-hazard model to compare the impact of CD15+ TINs and other clinical pathological parameters on the prognosis of the 115 patients in the training group. The univariate analysis identified that the CD15+ TINs (p = 0.003), the tumor size (p = 0.017), the depth of invasion (p = 0.010), the lymph node metastasis (p<0.001) and the distance metastasis (p<0.001) were significantly associated with overall survival (Table 2). Results from the multivariate analysis showed that the CD15+ TINs (p = 0.027), the depth of invasion (p = 0.041), the lymph node metastasis (p = 0.015) and the distance metastasis (p = 0.040) were independent prognostic factors of overall survival (Table 2). The relative risk in patients with high levels of CD15+ TINs was 1.943 times greater than that of patients with low levels of CD15+ TINs. Therefore, the level of CD15+ TINs may predict the overall survival in patients with gastric adenocarcinoma.

**Table 2:** Univariate and multivariate analysis of overall survival in gastric adenocarcinoma patients in the training group.

Variables	Univariate analysis			Multivariate analysis		
	HR	95% CI	*p* value	HR	95% CI	*p* value
CD15+TINs (lowvhigh)	2.296	1.317–4.001	0.003^a^	1.943	1.079–3.497	0.027^a^
Age (<60 v≧60)	1.258	0.744–2.127	0.846			
Gender (malevfemale)	0.687	0.522–1.525	0.676			
Tumor size (<4 cmv≧4 cm)	2.392	1.170–4.890	0.017^a^	1.068	0.491–2.324	0.869
Depth of invasion(T1-T2vT3-T4)	6.402	1.559–26.289	0.010^a^	4.529	1.065–19.263	0.041^a^
Lymph node metastasis(N0vN1-N4)	4.756	2.147–10.538	<0.001^a^	2.973	1.234–7.162	0.015^a^
Distance metastasis(novyes)	4.032	0.791–2.551	<0.001^a^	2.306	1.039–5.118	0.040^a^
Histologic grade(well/moderate/poor)	1.079	0.782–1.489	0.644			

*HR* Hazard ratio, *CI* confidence interval.

a *p* value<0.05.

### Validation of CD15+ TINs for survival prediction in the test group

**Figure 3:**
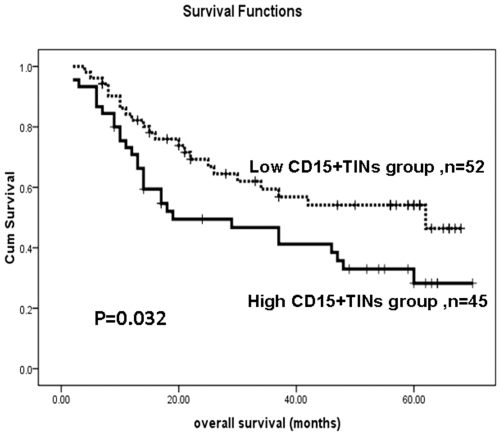
The Kaplan–Meier survival analysis of gastric adenocarcinoma patients (n = 97) in the test group with a low density of CD15+ TINs (n = 52) and a high density of CD15+ TINs (n = 45). The patients were divided into high CD15+ TINs group (≧21.60 cells/HPF) and low CD15+ TINs group (<21.60 cells/HPF) according to the median density of CD15+ TINs (21.60 cells/HPF), which was obtained from the training group. Log-rank test shows that the patients with the high density of CD15+ TINs showed significantly poorer prognosis than those with the low density of CD15+ TINs (p = 0.032).

To validate CD15+TINs for prediction survival of gastric adenocarcinoma patients, another 97 patients who underwent surgical resection in the same hospital during 2002 to 2005 were used as a test group. The 97 patients were divided into high CD15+ TINs group (n = 45, ≧ 21.60 cells/HPF) and low CD15+ TINs group (n = 52, <21.60 cells/HPF) based on the median density of CD15+ TINs (21.60 cells/HPF), which was obtained from the training group. Kaplan-Meier survival analysis on the patients with high CD15+ TINs infiltrating and low CD15+ TINs infiltrating also was conducted as did in the training group. The additional experiments results also showed that the patients with the high density of CD15+ TINs were markedly poorer than those of patients with the low density of CD15+ TINs. (Fig. 3, p = 0.032, long rank test).

## Discussion

In the current study, we performed the immunohistochemical analysis to determine the CD15 expression, which is a marker of neutrophils in gastric adenocarcinoma. We investigated the interrelation of the level of CD15+ TINs and the clinicopathological parameters in gastric adenocarcinoma. The CD15+ TINs were significantly associated with UICC staging (p = 0.028), lymph node metastasis (p = 0.024) and distance metastasis (p = 0.004) (Table 1). Therefore, we believed that the CD15+ TINs are more likely to contribute to tumor growth, progression and metastasis. The mechanism has not been elucidated, and there are few directed reports that address the mechanism. We hypothesize TINs may promote the migratory activity of tumor cells. Carina et al. have shown that neutrophil granulocytes induced intracellular signaling in tumor cells through ICAM-1 clustering and promoted the migratory activity of MDA-MB-468 human breast carcinoma cells [13]. Another possible mechanism is through angiogenesis. Kuang et al. showed that high infiltration of peritumoral neutrophils was positively correlated to angiogenic progression at the tumor-invading edge in HCC patients. The researchers also found that selective depletion of neutrophils effectively inhibited tumor angiogenesis and growth in vivo [20]. Infiltrating neutrophils also played a crucial role in activating angiogenesis in previously quiescent vasculature during the early stages of carcinogenesis [7], [21]. Although the results from the current study show a correlation between the number of CD15+ TINs and overall survival, the mechanism of TIN action to mediate survival remains to be elucidated.

The Kaplan–Meier survival analysis revealed that the high level of CD15+ TINs significantly correlated to the shorter survival time of gastric adenocarcinoma patients (Fig. 2, p = 0.002, long rank test). The result of high level of CD15+ TINs significantly associated with poor prognosis was also validated in the test group (Fig. 3, p = 0.032, long rank test). Furthermore, the univariate analysis indicated that the level of CD15+ TINs was a significant risk factor (HR = 2.296, p = 0.003) that affected the overall survival of gastric adenocarcinoma patients. The multivariate analysis demonstrated that the level of CD15+ TINs was an independent predictor (HR = 1.943, p = 0.027) of overall survival (OS) in gastric adenocarcinoma patients (Table 2). These results suggest that CD15+ TINs may be a novel prognostic indicator for gastric cancer. Coincident with our results, Hanne Krogh Jensen et al. reported that the presence of intratumoral neutrophils was as a poor independent prognostic factor for OS following nephrectomy in localized clear cell RCC [19]. Li et al. recently determined that intratumoral neutrophils were a poor prognostic factor for hepatocellular carcinoma following resection [22]. Furthermore, elevated neutrophil and monocyte counts in peripheral blood are associated with poor survival in patients with metastatic melanoma [23]–[24], small lung cell carcinoma [25], as well as gastric cancer [26]–[28]. The results from these studies support our current observations. In various carcinomas, neutrophils in the blood and located in the tumor are poor prognostic features. These results indicate that neutrophils play a negative role in various cancers and may be a target for intervenient therapy.

In conclusion, we evaluated the prognostic significance of the CD15+ TINs in a large number of clinical tissue specimens of gastric adenocarcinoma by immunohistochemical analysis in the current study. High level of CD15+ TINs in gastric adenocarcinoma patients correlated to disease progression and poor clinical outcome. CD15+ TINs was identified as an independent prognostic factor for overall survival in patients with gastric adenocarcinoma. Our findings suggest that CD15+ TINs is a novel prognostic marker for gastric adenocarcinoma.

## Materials and Methods

### Patients and tissue specimens

Formalin-fixed, paraffin-embedded tissues were obtained from gastric adenocarcinoma patients who underwent surgical resection at the Sun Yat-sen University Cancer Center between 2002 and 2005. In the gastric adenocarcinoma patients, 115 cases were assigned as the training group and 97 cases were assigned as the test group. These patients did not receive preoperative chemotherapy or radiotherapy. Patients with autoimmune diseases were excluded. Each tumor sample was assigned a histological grade based on the World Health Organization (WHO) classification criteria. All patients were staged using the 7th edition of the International Union Against Cancer (UICC) Tumor-Node-Metastasis (TNM) staging system. The follow-up dates of the patients in this study are available and complete. Postoperative follow-up occurred at our outpatient department and included clinical and laboratory examinations every 3 months for the first 2 years, every 6 months during the third to fifth years, annually for an additional 5 years or until patient death, whichever occurred first. Overall survival, which was defined as the time from the operation to the patient's death or the last follow-up, was used as a measure of prognosis. The characteristics of the gastric adenocarcinoma patients are shown in Table 3. All samples were coded anonymously in accordance with local ethical guidelines (as stipulated by the Declaration of Helsinki). The study was approved by the Ethics Committee of Sun Yat-sen University Cancer Center, and written informed consent was obtained from each patient.

**Table 3:** Clinical characteristics of gastric adenocarcinoma patients in the training group and test group.

Characteristics	Training Group (%)	Test Group (%)	*p* value
**Age(years)**			0.090
<60	59 (51.3)	61 (62.9)	
≧60	56 (48.7)	36 (37.1)	
**Gender**			0.496
Male	74 (64.3)	58 (59.8)	
Female	41 (35.7)	39 (40.2)	
**Tumor size(cm)**			0.737
<4	32 (27.8)	25 (25.8)	
≧4	83 (72.2)	72 (74.2)	
**Depth of invasion**			0.675
T1	5 (4.4)	3 (3.1)	
T2	12 (10.4)	6 (6.2)	
T3	25 (21.7)	23 (23.7)	
T4	73 (63.5)	65 (67.0)	
**Lymph node metastasis**			0.734
N0	37 (32.2)	25 (25.8)	
N1	20 (17.4)	21 (21.6)	
N2	24 (20.9)	21 (21.6)	
N3	34 (29.5)	30 (31.0)	
**Distance metastasis**			0.075
M0	107 (93.0)	83 (85.6)	
M1	8 (7.0)	14 (14.4)	
**UICC staging**			0.366
I	13 (11.3)	7 (7.2)	
II	30 (26.1)	23 (23.7)	
III	63 (54.8)	53 (54.7)	
IV	9 (7.8)	14 (14.4)	
**Histologic grade**			0.046
Well	27 (23.5)	14 (14.4)	
Moderate	29 (25.2)	17 (17.5)	
Poor	59 (51.3)	66 (68.1)	
**Death**			0.573
No	59 (51.3)	46 (47.4)	
Yes	56 (48.7)	51 (52.6)	

### Immunohistochemistry

The paraffinic-embedded tissue blocks were sectioned at a thickness of 2 µm for immunohistochemistry. The sections were deparaffinized and rehydrated using graded ethanol. For the antigen retrieval, the slides were immersed in EDTA (1 mmol/L, pH 8.0) and boiled for 15 minutes in a microwave oven. After rinsing with PBS, endogenous peroxidase was blocked with 3% hydrogen peroxide for 15 minutes at room temperature. The slides were incubated with the primary mouse anti-CD15 monoclonal antibody (Zhongshan Golden Bridge Biotech, Beijing, China) using a 1∶100 dilution in a moist chamber at 4°C overnight. After the primary antibody incubation, the slides were washed with PBS three times. The sections were incubated with horseradish peroxidase-conjugated secondary antibody (Zhongshan Golden Bridge Biotech, Beijing, China) for 30 minutes at room temperature. Following this incubation, the slides were washed three times in PBS. Finally, 3, 3′-diaminobenzidine tetrahydrochloride (DAB) was used to visualize the signal development, and then, the sections were counterstained with 20% hematoxylin. The analysis was performed by two independent observers using a Leica DM IRB inverted research microscope (Leica Microsystems, Wetzlar, Germany) [29]. The tumor sections were screened at low power magnification (100×), and the ten most representative fields were selected [29]. In order to evaluate the density of CD15+ TINs, each respective area of the tumor nest was manually measured at 400× high power magnification [10], [12], [29]. The density of stained cells was determined by computing the mean number of positively stained cells per high powermicroscopic field (HPF) [10], [12], [29]. Based on the median number of CD15+ TINs in the training group, the patients were separated into high and low TINs groups. Each sample was incubated using the same isotype antibody dilution under the same experimental conditions as the negative control.

### Statistical Analysis

Quantitative values were expressed as the mean ± SD or median (range). The median value was used as a cut-off for the subgroups of all immunohistochemical variables in our data. Chi-squared tests were used to assess the relationship between CD15+ TINs and the clinicopathological features. Overall survival (OS) was defined as the period from the initial diagnosis to death by any cause. Overall survival curves were calculated by the Kaplan-Meier method and analyzed by the long-rank test. Prognostic factors were examined by univariate and multivariate analyses using a Cox proportional hazards model. A two-sided *p*-value<0.05 was considered statistically significant. All statistical analyses were performed using SPSS software (version 16.0; SPSS Inc., Chicago, IL, USA).
